# Multimodal Cancer Therapy and Accelerated Brain Aging: Mechanisms, Biomarkers, and Clinical Consequences

**DOI:** 10.3390/curroncol33020121

**Published:** 2026-02-18

**Authors:** Mark Voynov, Maria Pospelova, Alexandra Nikolaeva, Varvara Krasnikova, Albina Makhanova, Olga Fionik, Konstantin Samochernykh, Tatyana Alekseeva, Stephanie E. Combs, Maxim Shevtsov

**Affiliations:** 1 Almazov National Medical Research Centre, Akkuratova Str. 2, 197341 Saint Petersburg, Russia; pospelovaml@mail.ru (M.P.); shura.nicolaeva@yandex.ru (A.N.); krasnikova_vv@almazovcentre.ru (V.K.); a.mahanova.a@mail.ru (A.M.); fvolga@mail.ru (O.F.); neurobaby12@gmail.com (K.S.); t.alekseeva@mail.ru (T.A.); 2 Department of Radiation Oncology, Technishe Universität München (TUM), Klinikum rechts der Isar, Ismaninger Str. 22, 81675 Munich, Germany; stephanie.combs@tum.de; 3 Laboratory of Biomedical Nanotechnologies, Institute of Cytology of the Russian Academy of Sciences (RAS), Tikhoretsky Ave., 4, 194064 Saint Petersburg, Russia

**Keywords:** chemotherapy, radiotherapy, targeted therapy, cancer-related cognitive impairment, accelerated brain aging, cognitive dysfunction, cancer survivorship

## Abstract

Advances in cancer therapy have markedly improved survival rates; however, long-term neurological sequelae represent a significant clinical challenge. Cancer treatment-related cognitive impairment (CRCI), commonly referred to as “chemobrain”, affects a substantial proportion of cancer survivors and encompasses a broad spectrum of neuropsychiatric and cognitive symptoms, including anxiety, depression, fatigue, balance disturbances, and deficits in attention, memory, processing speed, and executive function. Increasing evidence suggests that these manifestations reflect accelerated biological aging of the brain, rather than merely transient toxic effects. This review synthesizes current clinical, molecular, and neuroimaging evidence supporting the concept of accelerated brain aging associated with multimodal cancer therapy. We summarize key molecular and cellular mechanisms including oxidative stress, neuroinflammation, blood–brain barrier dysfunction, mitochondrial impairment, cellular senescence with a senescence-associated secretory phenotype, and epigenetic remodeling that overlap with physiological brain aging hallmarks. Particular attention is given to circulating molecular biomarkers of accelerated aging, such as inflammatory mediators, senescence markers, endothelial and neuronal injury indicators, and epigenetic age acceleration, and their potential translational relevance. We discuss clinical and neuropsychological data alongside structural and functional magnetic resonance imaging findings demonstrating cortical thinning, altered gyrification, white matter microstructural changes, disrupted functional connectivity, and increased brain age estimates following cancer therapy. Framing CRCI within an accelerated brain aging paradigm may improve risk stratification, guide biomarker development, and inform personalized survivorship care.

## Simple Summary

Cancer therapies have improved survival, but many survivors develop long-term cognitive and neurological problems known as cancer treatment-related cognitive impairment (CRCI or “chemobrain”). Symptoms include memory and attention deficits, fatigue, mood changes, and balance problems. Growing evidence suggests these effects reflect accelerated brain aging rather than temporary toxicity. This review summarizes clinical, molecular, and neuroimaging data showing that cancer therapy induces processes similar to normal brain aging, including oxidative stress, inflammation, mitochondrial dysfunction, cellular senescence, and epigenetic changes. Brain imaging reveals structural and functional alterations, while circulating biomarkers indicate aging-related damage. Viewing CRCI as accelerated brain aging may improve diagnosis, biomarker development, and personalized rehabilitation strategies.

## 1. Introduction

Cancer remains one of the leading global causes of morbidity and mortality in the 21st century. According to the most recent International Agency for Research on Cancer GLOBOCAN estimates, there were nearly 20 million new cancer cases worldwide in 2022, alongside approximately 9.7 million cancer-related deaths [1]. In the United States alone, an estimated 2.04 million new cancer cases are projected to be diagnosed in 2025, with approximately 618,120 cancer-related deaths [2]. These statistics underscore the enormous and growing burden of cancer, driven by aging populations, persistent exposure to risk factors such as tobacco use, unhealthy diets, environmental carcinogens, and infectious agents worldwide [3,4].

The therapeutic landscape for cancer is inherently multimodal. Surgical resection and radiation therapy are foundational for locoregional disease control and potentially curative interventions in many solid tumors [5,6]. Radiotherapy remains an integral component of multimodal cancer therapy and contributes substantially to improved survival across multiple tumor entities [7]. Meanwhile, systemic therapies, including chemotherapy, targeted agents, and immunotherapies, play indispensable roles in treating both localized and metastatic disease [8,9]. The potential of emerging therapeutic approaches, such as gene therapy, is also being actively investigated [10].

Chemotherapy continues to be a cornerstone of systemic treatment, utilizing cytotoxic agents that preferentially target rapidly dividing cells to reduce tumor burden and improve survival outcomes across numerous malignancies [11,12]. Chemotherapeutic regimens include alkylating agents, antimetabolites, topoisomerase inhibitors, and microtubule-destabilizing drugs, often administered in combination to enhance antitumor efficacy and mitigate resistance [13]. Given the systemic nature of chemotherapeutic agents, selecting an appropriate dosing regimen is critically important, and therapeutic drug monitoring is increasingly employed to optimize efficacy while minimizing toxicity [14]. However, certain malignancies, such as small-cell lung cancer, exhibit intrinsic or rapidly acquired resistance to many chemotherapy regimens, limiting their long-term therapeutic efficacy [15]. Over the past two decades, significant advancements in cancer therapy have included targeted therapies, such as tyrosine kinase inhibitors and monoclonal antibodies targeting specific molecular pathways, and immune checkpoint inhibitors, which harness the patient’s own immune system to recognize and eradicate tumor cells [16].

Nevertheless, the success of cancer therapies is tempered by their adverse effects. Beyond classical toxicities such as myelosuppression, gastrointestinal mucositis, alopecia, and fatigue, both the disease itself and its treatment can give rise to complex and long-lasting sequelae that significantly impact survivors’ quality of life [17]. Acute complications may involve organ dysfunction and systemic inflammation, while chronic sequelae can persist long after therapy completion [18]. Among breast cancer (BC) survivors, these include neurological, lymphatic, and biomechanical disturbances that may emerge months or years post-treatment [19]. Cancer treatment exerts damaging effects on both the peripheral and central nervous systems (CNS) [20,21,22]. The most pronounced symptoms appear in women undergoing combined surgery, chemotherapy and radiotherapy [23]. The severity of these disorders correlates with late-stage diagnosis of BC and concurrent chemotherapy [24].

Among these, cancer treatment-related cognitive impairment (CRCI) commonly referred to as “chemobrain” or “chemofog” has emerged as a clinically significant and frequently reported complication, particularly among patients receiving chemotherapy. The term “chemobrain” was introduced in the early 2000s to describe cognitive impairments associated with chemotherapy [25]. CRCI encompasses a range of cognitive disturbances—including impairments in memory, attention, processing speed, and executive function—that can persist months or years post-therapy and contribute to reduced daily functioning, employment challenges, and psychological distress in survivors [26]. Additionally, immune, targeted, endocrine, and radiotherapy modalities have also been implicated in cognitive impairment, suggesting that treatment-related effects may arise from diverse mechanisms associated with multimodal cancer therapy [27]. While CRCI manifestations have been increasingly documented, the underlying biological mechanisms remain incompletely understood and are the subject of intensive ongoing research.

Given this wide spectrum of adverse effects of cancer therapies on the central nervous system, an important question arises regarding the long-term consequences of such exposures on brain structure and function. Specifically, it remains unclear whether therapy-induced neural injury represents a transient, treatment-related phenomenon or whether anticancer therapies trigger or accelerate fundamental biological processes associated with normal brain aging. This perspective shifts emphasis from isolated cognitive side effects to a broader framework in which cancer therapy is considered a potential modifier of the brain’s biological aging trajectory.

Normal brain aging is a complex, multifactorial process that affects neural structure and function across multiple scales, from subcellular mechanisms to organ-level morphology. Morphologically, brain aging is most prominently characterized by progressive cerebral atrophy, including loss of total brain volume, cortical thinning, widening of sulci, reduction in gyrification, and enlargement of the ventricles, changes that have been consistently demonstrated in cross-sectional and longitudinal neuroimaging studies of cognitively normal adults [28]. At the cellular level, physiological brain aging features mitochondrial dysfunction, increased oxidative stress, impaired proteostasis, altered calcium homeostasis, and reduced DNA repair efficiency. Neurons exhibit age-related shrinkage and dendritic arbor simplification, rather than widespread cell loss, while synaptic density and plasticity decline, particularly in memory and executive function regions [29]. Concurrently, age-dependent glial changes contribute substantially to neural aging: microglia adopt a primed, pro-inflammatory phenotype; astrocytic support dysregulates; and oligodendrocytes exhibit diminished myelin maintenance, collectively impairing network integrity and signal transmission [30].

Cancer therapy acts as a systemic and local biological stressor that induces molecular, cellular, and vascular alterations overlapping with physiological aging mechanisms, thereby potentially accelerating the brain’s biological aging trajectory beyond transient neurotoxicity. An unresolved challenge is determining where normal age-related changes end and treatment-induced accelerated brain aging begins.

Cancer represents a major and escalating global health burden with profound implications for patients’ survival and long-term quality of life. While advancements in diagnosis and treatment have improved outcomes for many malignancies, the long-term consequences of both the disease and its therapies, particularly on cognitive health, remain significant. Understanding how cancer therapies intersect with physiological brain aging processes is essential for elucidating mechanisms of CRCI and for developing interventions to mitigate these effects.

## 2. Molecular and Cellular Mechanisms of Chemotherapy-Induced Brain Aging

The primary targets of antineoplastic drugs are rapidly dividing cells, such as those of the bone marrow and gastrointestinal tract. Although neurons do not rapidly proliferate, chemotherapeutic agents can still affect them both directly (via direct neurotoxic effects) and indirectly (via glial damage and neuroinflammation).

### 2.1. Oxidative Stress and Mitochondrial Dysfunction

One of the most consistently identified mechanisms of chemotherapy effects on the brain is oxidative stress, extensively studied in neurodegenerative diseases such as Alzheimer’s disease [31]. Many chemotherapeutic drugs, including platinum-based compounds, anthracyclines (e.g., doxorubicin), and taxanes, generate excessive reactive oxygen species (ROS) to target rapidly dividing tumor cells [32]. However, these ROS also damage neuronal and glial cells, which have inherently lower antioxidative capacities than other tissues. The brain’s high metabolic rate and reliance on oxidative phosphorylation further heighten susceptibility to oxidative damage [33].

Elevated ROS leads to lipid peroxidation, protein oxidation, and DNA damage, overwhelming endogenous antioxidant defenses and causing mitochondrial dysfunction. When ROS exceeds buffering capacity, mitochondrial membranes become permeabilized, impairing ATP synthesis, disrupting calcium homeostasis, and activating apoptotic pathways. This cascade amplifies oxidative injury, promoting neuronal apoptosis or senescence [34].

### 2.2. Neuroinflammation and Cytokine Signaling

Chemotherapy elicits a neuroinflammatory response that contributes to accelerated aging phenotypes in the brain. Systemic release of pro-inflammatory cytokines follows cytotoxic treatment. These cytokines can cross the blood–brain barrier (BBB) or affect the barrier’s permeability, thereby promoting microglial activation and a chronic inflammatory state within the CNS [35].

Emerging evidence suggests that adenosine signaling, particularly through the A_2A_ receptor, may modify neuroinflammatory responses and synaptic function after chemotherapy, indicating a potential mechanistic link between altered purinergic signaling and the accelerated aging-like cognitive sequelae observed in treated patients [36].

### 2.3. Blood–Brain Barrier Dysfunction

The BBB regulates CNS homeostasis, restricting the entry of peripheral toxins and inflammatory factors. Many chemotherapeutic agents compromise BBB integrity either directly (via endothelial toxicity) or indirectly (via systemic inflammation and cytokines) [33]. A disrupted BBB allows peripheral pro-inflammatory cytokines and ROS to enter the brain parenchyma more freely, exacerbating neurotoxic cascades and ultimately leading to neuronal injury [37]. Preclinical models confirm treatment-associated BBB permeability increases and subsequent cognitive deficits [38].

### 2.4. Impaired Neurogenesis and Synaptic Plasticity

Adult neurogenesis, particularly in the hippocampus, underpins learning and memory. Chemotherapy impairs neural progenitor proliferation and differentiation, especially in the hippocampal dentate gyrus. Rodent models show that methotrexate, 5-fluorouracil, and similar agents reduce hippocampal neurogenesis, correlating with persistent spatial memory and executive function deficits [39].

These effects also diminish synaptic plasticity, including long-term potentiation, essential for memory consolidation. Neurotransmission is altered via direct drug effects on neurotransmitter systems or indirectly through inflammation and oxidative stress [32].

### 2.5. DNA Damage and Disrupted Repair Mechanisms

Many antineoplastic drugs exert their anti-tumor effects by inducing DNA damage to halt the proliferation of cancer cells [40]. However, this mechanism is not selective solely to malignant cells; neuronal DNA can also accumulate damage when exposed to circulating cytotoxic drugs. Chemotherapy-induced DNA adducts, strand breaks, and oxidative lesions can overwhelm repair pathways, resulting in persistent DNA damage responses that promote cellular senescence, apoptosis, or impaired cellular function [35].

### 2.6. Cellular Senescence and SASP-Mediated Effects

Chemotherapy triggers cellular senescence in both dividing and non-dividing cells via DNA damage, oxidative stress, or telomere dysfunction. Senescent cells secrete a variety of pro-inflammatory and matrix-modifying factors collectively known as the senescence-associated secretory phenotype (SASP). The SASP perpetuates local inflammation, perturbs tissue microenvironments, and spreads senescence to neighboring cells, further accelerating age-related decline in brain tissues [41]. The role of the SASP is also prominent in Parkinson’s disease [42].

### 2.7. Epigenetic Alterations and Aging Pathways

Chemotherapy induces epigenetic modifications mirroring natural aging: altered DNA methylation, histone changes, and non-coding RNA expression [40]. Such reprogramming affects gene expression related to synaptic function, stress responses, and immune signaling, contributing to long-term cognitive and neural resilience deficits [43]. Studies show cancer therapies, including chemotherapy, are associated with accelerated epigenetic aging [44,45]. In early-stage breast cancer patients, adjuvant chemotherapy induces significant epigenetic age acceleration post-treatment, with partial persistence on follow-up [46]. These suggest chemotherapy shifts systemic aging trajectories, influencing CNS aging through immune-metabolic and inflammatory pathways [47].

**Figure 1:**
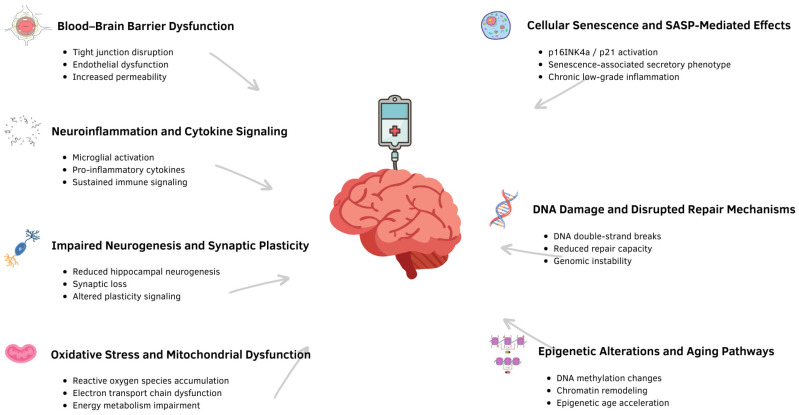
Key molecular and cellular pathways underlying chemotherapy-induced brain aging. This figure was created using Canva (Canva Pty Ltd., Sydney, Australia, 2026).

Taken together, these mechanisms illustrate how chemotherapy can accelerate biological aging processes within the brain through convergent cellular pathways: enhanced oxidative stress, chronic neuroinflammation, impaired BBB integrity, reduced neurogenesis, accumulated DNA and mitochondrial DNA damage, cellular senescence with SASP amplification, and persistent epigenetic drift. Many overlap with normative brain aging and neurodegenerative disorders, suggesting chemotherapy propels the brain toward an aged or pathological phenotype faster than chronological aging alone. An overview of these major molecular and cellular pathways in chemotherapy-induced brain aging is summarized in Figure 1.

## 3. Biomarkers of Chemotherapy-Induced Brain Aging

Identifying molecular biomarkers of accelerated brain aging post-chemotherapy is crucial for elucidating CRCI mechanisms and developing predictive tools for vulnerable patients. These biomarkers offer insights into systemic processes such as inflammation, oxidative stress, endothelial dysfunction, cellular senescence, epigenetic remodeling, and metabolic dysregulation, which influence CNS integrity. Crucially, many overlap with hallmarks of physiological aging and neurodegeneration, reinforcing the view that chemotherapy accelerates biological brain aging rather than causing isolated neurotoxicity [41].

Among the most consistently reported biomarker changes in cancer survivors are markers of chronic low-grade inflammation [48]. Chemotherapy induces both acute and persistent immune activation, resulting in sustained elevations of circulating pro-inflammatory mediators [49]. Increased serum levels of interleukin-6 (IL-6), tumor necrosis factor-α (TNF-α), interleukin-1β (IL-1β), and C-reactive protein (CRP) have been consistently reported in cancer survivors after systemic therapy and have been associated with fatigue, cognitive complaints, and reduced quality of life [32,41,50]. Persistent systemic inflammation may affect brain aging indirectly by promoting BBB dysfunction, microglial activation, and synaptic dysregulation, thereby linking peripheral immune markers to central aging processes [51].

Oxidative stress biomarkers provide another important molecular window into therapy-associated brain aging. Chemotherapeutic agents are potent inducers of ROS, and biomarkers reflecting oxidative damage have been detected long after treatment completion [52]. Elevated levels of lipid peroxidation products (malondialdehyde, 4-hydroxynonenal), oxidized DNA bases (8-hydroxy-2′-deoxyguanosine), accompanied by reduced antioxidant defenses such as glutathione, have been reported in chemotherapy-treated patients [53].

Epigenetic biomarkers have emerged as robust indicators of biological aging. DNA methylation-based epigenetic clocks estimate biological age independently of chronological age and predict age-related morbidity and mortality [51]. Although epigenetic clocks are derived from peripheral blood, their strong associations with cognitive decline and neurodegenerative risk suggest potential relevance for brain aging through immune-metabolic and inflammatory pathways.

Cellular senescence-associated biomarkers further strengthen the link between chemotherapy and accelerated aging biology. Senescent cells secrete a SASP characterized by increased circulating levels of IL-6, Interleukin-8 (IL-8), Monocyte chemoattractant protein-1 (MCP-1), and Matrix metalloproteinases (MMPs), which propagate chronic inflammation and tissue dysfunction [54]. Markers of senescence, including increased expression of p16^INK4a^ and elevated circulating SASP components, have been proposed as biomarkers of accelerated biological aging following chemotherapy [55].

Our research group’s studies provide direct empirical evidence supporting the utility of circulating molecular biomarkers reflecting CNS and endothelial injury in cancer survivors. In breast cancer patients with neurological complications following therapy, elevated serum levels of biomarkers associated with neuronal and endothelial damage were observed and linked to systemic treatment effects [56,57]. Specifically, increased circulating levels of intercellular adhesion molecule-1 (ICAM-1) and platelet endothelial cell adhesion molecule-1 (PECAM-1) were consistently detected in breast cancer survivors after comprehensive oncologic treatment, indicating persistent endothelial activation and systemic inflammation [58]. These findings are supported by other studies. In a human cerebral microvascular endothelial cell model, Patel et al. reported that doxorubicin exposure significantly increased intercellular adhesion molecule-1 expression alongside other proinflammatory mediators [59]. Endothelial dysfunction is a recognized contributor to vascular aging and may indirectly influence brain aging by impairing neurovascular homeostasis.

Neurotrophic factors, particularly brain-derived neurotrophic factor (BDNF), have also emerged as potential biomarkers linking CRCI with biological brain aging. Evidence suggests that reduced BDNF levels may contribute to CRCI, while interventions aimed at enhancing BDNF signaling are being explored as potential therapeutic strategies [60,61]. Recent evidence further suggests a relationship between BDNF levels and epigenetic age acceleration, with studies in younger cancer populations highlighting the potential clinical relevance of this association [62]. Synaptic function-related biomarkers are also gaining attention in CRCI research. Dynamin-1, a key regulator of synaptic vesicle endocytosis and neurotransmission, has been implicated in chemotherapy-associated cognitive impairment, supporting the contribution of synaptic dysfunction to therapy-related brain aging processes [63].Neuron-specific enolase (NSE), a peripheral indicator of neuronal injury and metabolic stress, was elevated in treated patients, reflecting ongoing neural stress after therapy [64]. Claessens et al. confirm NSE as a peripheral biomarker of central nervous system injury in patients with ifosfamide-induced encephalopathy [65]. In addition, altered levels of anti-N-methyl-D-aspartic acid receptor 2 (NR-2) antibodies, targeting the NR2 subunit of the NMDA receptor, were observed, suggesting persistent molecular changes related to CNS integrity and excitotoxic vulnerability [64]. According to Hadjiagapiou et al., anti-NR2 antibodies may be associated with brain injury in the context of blood–brain barrier disruption [66].

Collectively, these findings demonstrate that chemotherapy is associated with long-lasting alterations in circulating biomarkers reflecting inflammation, endothelial dysfunction, and neuronal stress—processes that closely parallel molecular mechanisms of physiological and accelerated brain aging (Table 1).

The interpretation of molecular biomarkers of therapy-induced brain aging is subject to several limitations. Most available studies rely on peripheral blood markers, which may not directly reflect central nervous system-specific processes and are influenced by systemic inflammation, comorbidities, and concurrent treatments. Heterogeneity in cancer types, therapeutic regimens, timing of biomarker assessment, and cognitive evaluation further limits cross-study comparability. In addition, many biomarkers lack established specificity for brain aging, complicating differentiation between transient treatment-related effects and sustained acceleration of biological aging. Longitudinal studies integrating molecular, neuroimaging, and clinical endpoints are needed to validate biomarker utility and causal relevance.

## 4. Radiotherapy and Accelerated Brain Aging

Radiotherapy can influence brain aging through two partially overlapping routes: indirect, systemic effects when irradiation is delivered to extracranial targets, and direct effects when the brain (or structures within the head/neck region) is included in the radiation field. Although these contexts differ in dose distribution and the primary tissue exposed, both can converge on biological pathways that resemble canonical hallmarks of brain aging—particularly chronic inflammation, vascular dysfunction, oxidative stress, and therapy-induced cellular senescence.

### 4.1. Indirect Effects on the Brain During Extracranial Radiotherapy

Indirect effects of radiotherapy on the brain are considerably less well characterized in the literature than the direct effects of cranial irradiation. Even when the central nervous system is not directly irradiated, radiotherapy can produce systemic inflammatory and immune shifts capable of affecting the brain. Ionizing radiation induces damage-associated molecular patterns, cytokine release, and immune cell activation in irradiated tissues, which can propagate “bystander” effects in distant organs [69].

A key mechanistic bridge is the peripheral inflammatory milieu, which may influence brain aging through cytokine trafficking, endothelial activation, and altered neuroimmune communication. Radiation-induced immune and inflammatory reactions, particularly persistent changes in innate immune signaling, have been described as relevant to cognitive and neural outcomes, and these systemic effects are conceptually aligned with inflammatory pathways implicated in accelerated brain aging [70]. Experimental and translational work further supports that radiation combined with other modalities can modify behavioral and neuroinflammatory endpoints, reinforcing the plausibility of systemic-to-brain signaling even when irradiation is not delivered directly to the brain [71,72].

### 4.2. Direct Effects of Cranial Radiotherapy

When the brain is within the radiation field (e.g., primary brain tumors, brain metastases, head-and-neck fields with partial brain exposure), mechanistic evidence supports a multi-level injury cascade that overlaps strongly with aging biology. Comprehensive reviews describe neuroinflammation and reduced neurogenesis as central drivers of radiation-induced brain injury, with downstream consequences for network integrity and long-term tissue resilience [73,74].

Cranial irradiation activates microglia and astrocytes, can promote infiltration of peripheral immune cells, and may establish a chronic inflammatory state with features that mirror aging-associated microglial priming and sustained cytokine signaling. Importantly, this inflammation is not merely an acute response; persistent innate immune activation serves as a substrate for late tissue dysfunction, consistent with an accelerated aging model rather than transient toxicity [75]. Vascular hypotheses of late radiation injury highlight endothelial damage, vessel wall remodeling, ischemic vulnerability, and secondary white matter injury [76]. BBB disruption is frequently described as a key mechanistic event that can amplify neuroinflammation, alter CNS homeostasis, and increase susceptibility to secondary insults [77]. Reviews consistently emphasize demyelination, fiber bundle disruption, and glial dysfunction as downstream consequences of cranial irradiation. Damage to oligodendrocytes and their precursors reduces repair capacity and resembles age-related decline in myelin integrity and white matter resilience [75,78]. Ionizing radiation generates reactive oxygen species, induces oxidative injury, and produces DNA damage (including double-strand breaks) that can overwhelm repair pathways and promote long-term genomic instability [77]. These processes align with core mechanisms of biological aging, particularly when sustained or repeatedly induced over treatment courses. Radiotherapy is a potent inducer of cellular senescence in both tumor and normal tissues. Senescent cells can persist and generate a SASP, characterized by pro-inflammatory cytokines and matrix-remodeling factors that sustain tissue dysfunction [79]. Recent evidence also points to activation of the complement system as a contributor to radiation-associated cognitive impairment. Experimental studies indicate that cranial irradiation can upregulate complement components such as C1q, C3 and C5, promoting microglial activation, synaptic remodeling, and persistent neuroinflammation [80,81].

Across both systemic and direct cranial exposure contexts, radiotherapy engages a convergent spectrum of biological pathways relevant to brain aging, including chronic neuroinflammation, vascular dysfunction, oxidative stress and DNA damage, impaired myelin maintenance, disrupted neuroplasticity, and therapy-induced cellular senescence. The convergence of these mechanisms supports the conceptualization of radiotherapy not merely as a source of localized tissue injury, but as a potential modifier of the brain’s biological aging trajectory. Notably, many of these pathways closely mirror those implicated in chemotherapy-associated central nervous system toxicity, underscoring shared mechanistic substrates through which distinct anticancer modalities may contribute to accelerated brain aging.

## 5. Targeted Therapy as a Modifier of Brain Aging

Targeted anticancer therapies, including small-molecule tyrosine kinase inhibitors and monoclonal antibodies, have reshaped oncology, yet accumulating evidence suggests that these agents can also be associated with cognitive and neuropsychiatric changes, raising the question of whether targeted therapy may contribute to aging-like alterations in brain biology [82]. A key challenge is that cognitive outcomes are often assessed in heterogeneous clinical contexts (advanced disease, prior chemotherapy, fatigue and sleep disturbance), but mechanistic frameworks increasingly emphasize that targeted agents can influence CNS homeostasis [83].

Targeted therapies can modulate cytokine networks directly (via pathway inhibition) or indirectly (through tumor-host interactions and systemic stress), and chronic low-grade inflammation is a central driver of physiological brain aging [48]. Additionally, the kinase pathways targeted in cancer overlap with processes required for synaptic plasticity, neuronal survival, glial function, and cellular stress responses [84]. While CNS exposure depends on drug properties and transporter-mediated efflux, clinical observations suggest that, at least in some patients, targeted agents may be associated with meaningful cognitive changes. In chronic myeloid leukemia cohorts treated with tyrosine kinase inhibitors, difficulty concentrating and memory complaints have been commonly endorsed, motivating systematic evaluation of cognition in this population [85]. Case-based evidence further supports plausibility: a detailed report described a strong temporal association between dasatinib therapy and neurocognitive impairment, with improvement after drug discontinuation, suggesting that individual vulnerability, off-target effects, or variable CNS exposure may contribute to targeted therapy-related cognitive toxicity [86]. Additional case literature describes cognitive and behavioral syndromes during tyrosine kinase inhibitor therapy that mimicked neurodegenerative presentations, again supporting the need to consider targeted agents as potential modifiers of brain function in selected individuals [87]. Emerging immune-based approaches such as chimeric antigen receptor T-cell (CAR-T) therapy and bispecific antibodies have also been associated with neurotoxicity, most notably immune effector cell-associated neurotoxicity syndrome, which is characterized by encephalopathy, cognitive dysfunction, and other neurological symptoms and is thought to involve inflammatory and endothelial mechanisms affecting central nervous system integrity [88,89].

Within breast cancer, anti-human epidermal growth factor receptor 2 (anti-HER2) therapy provides a focused example where cognitive effects have been specifically examined. A scoping review reported that several studies observed small-to-significant worsening of cognitive performance in regimens containing trastuzumab, while also noting that newer anti-HER2 antibody-drug conjugates may be associated with less cognitive impairment in some settings, underscoring regimen-specific and context-dependent effects [90]. Although causality is difficult to isolate because anti-HER2 agents are often delivered with chemotherapy, these findings support the broader mechanistic hypothesis that targeted therapies may contribute to cognitive vulnerability through network-level stressors overlapping with aging pathways, including vascular and inflammatory mechanisms [82].

Overall, the current literature supports a biologically plausible model in which targeted therapies may influence the brain’s aging trajectory. However, robust mechanistic attribution remains limited by heterogeneous cohorts and co-exposures, emphasizing the need for longitudinal studies integrating standardized cognitive testing with molecular aging biomarkers (e.g., senescence markers, inflammatory signatures, epigenetic age acceleration) to distinguish transient neurotoxicity from true acceleration of brain aging in cancer survivors.

## 6. Clinical Evidence of Cancer Treatment-Related Cognitive Impairment and Brain Aging

### 6.1. Clinical Manifestations and Cognitive Domains Affected by Cancer Treatment

Cancer treatment-related cognitive impairment describes a constellation of neurological and neuropsychiatric symptoms that cancer patients experience during and after systemic therapy, and it is increasingly recognized as a clinically meaningful syndrome rather than a subjective complaint. Epidemiological studies suggest that CRCI may affect a substantial proportion of cancer survivors. Up to 75% of patients report cognitive changes during chemotherapy, with approximately 30–35% experiencing persistent deficits months to years after treatment completion [91,92].

The clinical picture of “chemobrain” encompasses a broader range of symptoms beyond strictly cognitive domains. Anxiety and depressive symptoms are commonly reported in chemotherapy-treated patients and have been found to correlate with patients’ subjective cognitive concerns, even when objective measures vary [93,94]. Severe fatigue, sleep disturbances, and mood disorders frequently co-occur with cognitive complaints, compounding impairment in daily functioning [23,95,96]. These non-cognitive features may both exacerbate and mask underlying cognitive changes and are themselves linked to aging-related neurobiological processes. Vestibular symptoms such as dizziness and balance impairment have also been described, particularly in survivors exposed to neurotoxic agents, and are increasingly recognized in association with CRCI, especially in cohorts with concomitant physical complications such as chemotherapy-related peripheral neuropathy [97,98].

Objective assessment of cognitive performance using standardized neuropsychological tests reveals that cancer treatment can affect multiple cognitive domains [26]. The most commonly observed deficits involve:

- Processing speed and attention;
- Executive functions (e.g., planning, task switching);
- Working memory and short-term recall;
- Episodic memory.

Importantly, meta-analyses indicate that while the magnitude of these deficits is often small to moderate, the pattern of impairment closely parallels that seen in age-associated cognitive decline, particularly in frontal and hippocampal functions [99]. Moreover, while some patients demonstrate partial recovery over time, a subset exhibits persistent or even progressive impairment over the years post-treatment, suggesting that chemotherapy can accelerate cognitive aging trajectories in vulnerable individuals [92]. All described symptoms are summarized in Table 2.

According to recommendations of the International Cancer and Cognition Task Force, studies of CRCI should primarily focus on learning and memory, executive function, and processing speed/attention, which represent the cognitive domains most consistently affected in cancer survivors [100]. Risk factors for CRCI include older age, lower baseline cognitive reserve, comorbid vascular or metabolic disease, higher cumulative chemotherapy dosage, and combined modality therapies (e.g., chemotherapy plus radiotherapy) [101,102]. These risk profiles overlap with factors known to increase susceptibility to age-related cognitive decline, further supporting conceptual links between CRCI and accelerated brain aging.

Given the multifactorial mechanisms underlying CRCI, effective mitigation strategies are likely to require a multimodal and individualized approach. Emerging evidence supports the role of non-pharmacological interventions, including structured cognitive rehabilitation, aerobic and resistance exercise, and lifestyle modifications, in improving cognitive function and enhancing neuroplasticity in cancer survivors [103,104,105]. Several clinical trials are specifically evaluating interventions aimed at mitigating CRCI [106,107]. Randomized controlled studies of cognitive rehabilitation and cognitive training programs in cancer survivors have demonstrated improvements in attention, memory, and quality of life, supporting their potential role in survivorship care [104]. More recent trials are exploring web-based cognitive rehabilitation platforms, structured exercise interventions, and multimodal behavioral strategies designed to prevent or reduce CRCI during and after cancer therapy [108]. Pharmacological strategies targeting neuroinflammation, oxidative stress, and vascular dysfunction, such as anti-inflammatory agents, antioxidants, neuroprotective compounds, and cholinesterase inhibitors (e.g., donepezil) are under active investigation, although robust clinical evidence remains limited [20,26,109]. Importantly, early identification of individuals at increased risk through molecular biomarkers and neuropsychological assessment may enable timely preventive interventions. Integrating therapeutic and rehabilitative strategies into long-term survivorship care holds promise for attenuating accelerated brain aging and preserving cognitive health following chemotherapy.

### 6.2. Neuroimaging Evidence of Cancer Treatment-Related Cognitive Impairment

Neuroimaging provides objective evidence of treatment-related brain changes. Structural magnetic resonance imaging (MRI) can reveal acceleration of brain aging following chemotherapy, as demonstrated by longitudinal increases in predicted brain age and cortical thinning in breast cancer survivors [110]. Structural magnetic resonance imaging has also revealed alterations in cortical gyrification in patients who have undergone chemotherapy [111]. Diffusion-weighted MRI and diffusion tensor imaging provide sensitive measures of white matter microstructural integrity, which are altered after chemotherapy in patterns reminiscent of aging-related degeneration [112]. In one of our recent studies employing diffusion tensor imaging, microstructural white matter alterations in cerebellar and vestibular tracts were identified in breast cancer survivors with balance impairment in the long-term period after treatment, including decreased fractional anisotropy indicative of compromised axonal integrity. Notably, these changes were attenuated at later follow-up time points, indirectly suggesting the involvement of neuroplasticity-related processes [113]. In a study by Hyoung Seop Kim et al., cytarabine was shown to exert toxic effects on cerebellar structures in a patient with lymphoma. Although conventional magnetic resonance imaging revealed no structural abnormalities, brain positron emission tomography demonstrated diffuse hypometabolism with predominant involvement of the cerebellum [114]. Other chemotherapeutic agents associated with cerebellar dysfunction include capecitabine, 5-fluorouracil, hexamethylmelamine, nelarabine, oxaliplatin, procarbazine, and vincristine [115]. In rare cases, blinatumomab has also been reported to induce a cerebellar syndrome; in one cohort, this complication was observed in 2 of 95 patients and was reversible upon discontinuation of the drug [116]. Functional MRI further supports CRCI as a brain aging phenotype by demonstrating altered activation and connectivity patterns during cognitive tasks [117]. Using functional magnetic resonance imaging, Bukkieva et al. identified significant alterations in brain functional connectivity, with an overall reduction in connectivity in breast cancer survivors compared with healthy controls [118]. Magnetic resonance morphometric analyses have also demonstrated statistically significant reductions in the volumes of various brain structures in patients who have undergone treatment for breast cancer [119,120].

Together, neuropsychological and neuroimaging findings depict CRCI as a multifaceted clinical syndrome with objective neural correlates. Structural and functional brain changes in cancer survivors align with patterns observed in aging brains and may reflect accelerated biological processes involving neuroinflammation, vascular compromise, and impaired neurogenesis discussed in earlier sections.

## 7. Conclusions

Cancer treatment-related cognitive impairment represents a complex and clinically relevant consequence of oncologic therapy that extends beyond transient neurotoxicity. Accumulating molecular, clinical, and neuroimaging evidence supports the concept that multimodal cancer therapies may accelerate biological brain aging through mechanisms overlapping with physiological aging processes. Integrating molecular biomarkers, neuroimaging markers, and clinical assessment may improve identification of individuals at risk and inform personalized survivorship care. Future longitudinal and interventional studies are needed to delineate therapy-associated aging trajectories and to develop targeted strategies aimed at preserving cognitive function in cancer survivors.

## Figures and Tables

**Table 1:** Molecular biomarkers associated with accelerated brain aging following chemotherapy.

Biomarker Category	Representative Biomarkers	Biological Process Reflected	Key Findings in Chemotherapy-Treated Patients	References
Inflammatory biomarkers	IL-6, TNF-α, IL-1β, CRP	Chronic low-grade inflammation ("inflammaging")	Persistent elevation of pro-inflammatory cytokines after chemotherapy, associated with cognitive complaints and systemic aging phenotypes	[32,41,50,51,67]
Oxidative stress markers	Malondialdehyde, 4-hydroxynonenal, 8-hydroxy-2′-deoxyguanosine; ↓ glutathione	Oxidative damage and redox imbalance	Increased oxidative stress and reduced antioxidant capacity following chemotherapy, paralleling mechanisms of brain aging	[52,53,68]
Epigenetic aging markers	DNA methylation-based epigenetic clocks	Epigenetic age acceleration	Chemotherapy induces acceleration of epigenetic age relative to chronological age	[44,45,46,47]
Cellular senescence markers	p16^INK4a^, p21, IL-6, IL-8, MCP-1, MMPs	Therapy-induced cellular senescence	Increased senescence marker expression after cytotoxic therapy	[40,54,55]
Endothelial dysfunction markers	ICAM-1, PECAM-1	Endothelial activation, vascular inflammation, BBB vulnerability	Significantly increased circulating ICAM-1 and PECAM-1 levels in breast cancer survivors after therapy, indicating persistent endothelial dysfunction	[56,58,59]
Neuronal injury markers	NSE	Neuronal stress and metabolic injury	Altered NSE levels in chemotherapy-treated patients, reflecting ongoing neuronal stress	[64,65]
Autoantibodies to neuronal antigens	Anti-NR-2 antibodies	NMDA receptor-related neuronal alterations	Altered levels after therapy indicating long-term CNS changes	[64,66]
Neurotrophic biomarkers	BDNF	↓Synaptic plas-ticity, neurogene-sis, neuronal sur-vival	Reduced BDNF levels have been associated with cognitive im-pairment follow-ing chemothera-py	[60,61]
Synaptic function biomarkers	Dynamin-1	↓Synaptic vesicle trafficking, neurotransmission	Reduced Dynamin-1 levels has been associated with CRCI, suggesting synaptic dysfunction as a contributing mechanism	[63]

**Table 2:** Clinical Manifestations of Cancer Treatment-Related Cognitive Impairment.

Clinical Domain	Key Manifestations
Cognitive function	Impaired attention, reduced processing speed, executive dysfunction
Memory	Short-term and working memory impairment, reduced verbal and visuospatial recall
Executive function	Difficulties in planning, multitasking, cognitive flexibility, decision-making
Processing speed and attention	Slowed information processing, reduced sustained attention
Affective symptoms	Anxiety, depressive symptoms, emotional lability
Fatigue and sleep disturbances	Persistent fatigue, insomnia, altered sleep–wake cycle
Vestibular and balance symptoms	Dizziness, gait instability, impaired balance
Subjective cognitive complaints	Self-reported “brain fog”, forgetfulness, reduced mental clarity

## Data Availability

No new data were created or analyzed in this study.

## Abbreviations

The following abbreviations are used in this manuscript:

CNS	Central Nervous System
CRCI	Cancer Treatment-Related Cognitive Impairment
ROS	Reactive Oxygen Species
BBB	Blood–Brain Barrier
SASP	Senescence-Associated Secretory Phenotype
MRI	Magnetic Resonance Imaging
ICAM-1	Intercellular Adhesion Molecule-1
PECAM-1	Platelet Endothelial Cell Adhesion Molecule-1
NSE	Neuron-Specific Enolase
NR2	N-Methyl-D-Aspartate Receptor Subunit 2
IL-6	Interleukin-6
IL-8	Interleukin-8
TNF-α	Tumor Necrosis Factor-Alpha
CRP	C-Reactive Protein
BDNF	Brain-Derived Neurotrophic Factor
CAR-T	Chimeric Antigen Receptor T-cell
Anti-HER2	Anti-Human Epidermal Growth Factor Receptor 2

## References

[1] Bray F., Laversanne M., Sung H., Ferlay J., Siegel R.L., Soerjomataram I., Jemal A. (2024). Global Cancer Statistics 2022: GLOBOCAN Estimates of Incidence and Mortality Worldwide for 36 Cancers in 185 Countries. CA Cancer J. Clin..

[2] Siegel R.L., Kratzer T.B., Giaquinto A.N., Sung H., Jemal A. (2025). Cancer Statistics, 2025. CA Cancer J. Clin..

[3] Li T., Zhang H., Lian M., He Q., Lv M., Zhai L., Zhou J., Wu K., Yi M. (2025). Global Status and Attributable Risk Factors of Breast, Cervical, Ovarian, and Uterine Cancers from 1990 to 2021. J. Hematol. Oncol..

[4] Obeagu E.I., Obeagu G.U. (2024). Breast Cancer: A Review of Risk Factors and Diagnosis. Medicine.

[5] Fukata K., Akiyoshi T., Numao N., Komai Y., Mukai T., Hiyoshi Y., Yamaguchi T., Nagasaki T., Konishi T., Fukunaga Y. (2022). Robotic-Assisted Laparoscopic Surgery for Synchronous Primary Rectal and Prostate Cancer: Initial Case Series. Asian J. Endosc. Surg..

[6] Vendrely V., Rivin Del Campo E., Modesto A., Jolnerowski M., Meillan N., Chiavassa S., Serre A.A., Gérard J.P., Créhanges G., Huguet F. (2022). Rectal Cancer Radiotherapy. Cancer/Radiother..

[7] Escande A., Leblanc J., Hannoun-Levi J.M., Renard S., Ducassou A., Hennequin C., Chargari C. (2024). Place of Radiotherapy for Treatment of Metastatic Cervical, Vaginal and Endometrial Uterine Cancer. Cancer/Radiother..

[8] Jeon H., Wang S., Song J., Gill H., Cheng H. (2025). Update 2025: Management of Non-Small-Cell Lung Cancer. Lung.

[9] Heater N.K., Warrior S., Lu J. (2024). Current and Future Immunotherapy for Breast Cancer. J. Hematol. Oncol..

[10] Yoo H.C., Lee S., Park J.Y., Lee E.J. (2025). AAV for Ovarian Cancer Gene Therapy. Cancer Gene Ther..

[11] Soares do Brito J., Santos R., Sarmento M., Fernandes P., Portela J. (2023). Chemotherapy Regimens for Non-Metastatic Conventional Appendicular Osteosarcoma: A Literature Review Based on the Outcomes. Curr. Oncol..

[12] Massimo G., Fortunato M., Massimo M., Ernesto V., Enrica A.M., Francesco M., Giovanni M., Claudio C. (2021). Chemotherapy-Based Regimens in Multiple Myeloma in 2020. Panminerva Med..

[13] Mollaei M., Hassan Z.M., Khorshidi F., Langroudi L. (2021). Chemotherapeutic Drugs: Cell Death- and Resistance-Related Signaling Pathways. Are They Really as Smart as the Tumor Cells?. Transl. Oncol..

[14] Knezevic C.E., Clarke W. (2020). Cancer Chemotherapy: The Case for Therapeutic Drug Monitoring. Ther. Drug Monit..

[15] Ying Q., Fan R., Shen Y., Chen B., Zhang J., Li Q., Shi X. (2024). Small Cell Lung Cancer-An Update on Chemotherapy Resistance. Curr. Treat. Options Oncol..

[16] Ruiz-Cordero R., Devine W.P. (2020). Targeted Therapy and Checkpoint Immunotherapy in Lung Cancer. Surg. Pathol. Clin..

[17] Mahumud R.A., Shahjalal M., Dahal P.K., Mosharaf M.P., Hoque M.E., Wawryk O. (2024). Systemic Therapy and Radiotherapy Related Complications and Subsequent Hospitalisation Rates: A Systematic Review. BMC Cancer.

[18] Shahrokni A., Wu A.J., Carter J., Lichtman S.M. (2016). Long-Term Toxicity of Cancer Treatment in Older Patients. Clin. Geriatr. Med..

[19] Marco E., Trépanier G., Chang E., Mauti E., Jones J.M., Zhong T. (2023). Postmastectomy Functional Impairments. Curr. Oncol. Rep..

[20] Rao V., Bhushan R., Kumari P., Cheruku S.P., Ravichandiran V., Kumar N. (2022). Chemobrain: A Review on Mechanistic Insight, Targets and Treatments. Adv. Cancer Res..

[21] Bennedsgaard K., Grosen K., Attal N., Bouhassira D., Crombez G., Jensen T.S., Bennett D.L., Ventzel L., Andersen I.S., Finnerup N.B. (2022). Neuropathy and Pain after Breast Cancer Treatment: A Prospective Observational Study. Scand. J. Pain.

[22] Bae E.H., Greenwald M.K., Schwartz A.G. (2021). Chemotherapy-Induced Peripheral Neuropathy: Mechanisms and Therapeutic Avenues. Neurotherapeutics.

[23] Grusdat N.P., Stäuber A., Tolkmitt M., Schnabel J., Schubotz B., Wright P.R., Schulz H. (2022). Routine Cancer Treatments and Their Impact on Physical Function, Symptoms of Cancer-Related Fatigue, Anxiety, and Depression. Support. Care Cancer.

[24] Carreira H., Williams R., Dempsey H., Stanway S., Smeeth L., Bhaskaran K. (2021). Quality of Life and Mental Health in Breast Cancer Survivors Compared with Non-Cancer Controls: A Study of Patient-Reported Outcomes in the United Kingdom. J. Cancer Surviv..

[25] Wefel J.S., Lenzi R., Theriault R., Buzdar A.U., Cruickshank S., Meyers C.A. (2004). “Chemobrain” in Breast Carcinoma? A Prologue. Cancer.

[26] Onzi G.R., D’Agustini N., Garcia S.C., Guterres S.S., Pohlmann P.R., Rosa D.D., Pohlmann A.R. (2022). Chemobrain in Breast Cancer: Mechanisms, Clinical Manifestations, and Potential Interventions. Drug Saf..

[27] Fleming B., Edison P., Kenny L. (2023). Cognitive Impairment after Cancer Treatment: Mechanisms, Clinical Characterization, and Management. BMJ.

[28] Blinkouskaya Y., Caçoilo A., Gollamudi T., Jalalian S., Weickenmeier J. (2021). Brain Aging Mechanisms with Mechanical Manifestations. Mech. Ageing Dev..

[29] Lee J., Kim H.J. (2022). Normal Aging Induces Changes in the Brain and Neurodegeneration Progress: Review of the Structural, Biochemical, Metabolic, Cellular, and Molecular Changes. Front. Aging Neurosci..

[30] Gaspar-Silva F., Trigo D., Magalhaes J. (2023). Ageing in the Brain: Mechanisms and Rejuvenating Strategies. Cell. Mol. Life Sci..

[31] Ionescu-Tucker A., Cotman C.W. (2021). Emerging Roles of Oxidative Stress in Brain Aging and Alzheimer’s Disease. Neurobiol. Aging.

[32] Nguyen L.D., Ehrlich B.E. (2020). Cellular Mechanisms and Treatments for Chemobrain: Insight from Aging and Neurodegenerative Diseases. EMBO Mol. Med..

[33] Ren X., Boriero D., Chaiswing L., Bondada S., St. Clair D.K., Butterfield D.A. (2019). Plausible Biochemical Mechanisms of Chemotherapy-Induced Cognitive Impairment (“Chemobrain”), a Condition That Significantly Impairs the Quality of Life of Many Cancer Survivors. Biochim. Biophys. Acta Mol. Basis Dis..

[34] Cauli O. (2021). Oxidative Stress and Cognitive Alterations Induced by Cancer Chemotherapy Drugs: A Scoping Review. Antioxidants.

[35] Jaiswara P.K., Shukla S.K. (2023). Chemotherapy-Mediated Neuronal Aberration. Pharmaceuticals.

[36] Oliveros A., Poleschuk M., Cole P.D., Boison D., Jang M.H. (2023). Chemobrain: An Accelerated Aging Process Linking Adenosine A_2A_ Receptor Signaling in Cancer Survivors. Int. Rev. Neurobiol..

[37] Patai R., Csik B., Nyul-Toth A., Gulej R., Vali Kordestan K., Sai Chandragiri S., Shanmugarama S., Tarantini S., Mukli P., Ungvari A. (2025). Persisting Blood–Brain Barrier Disruption Following Cisplatin Treatment in a Mouse Model of Chemotherapy-Associated Cognitive Impairment. GeroScience.

[38] Csik B., Nyúl-Tóth Á., Gulej R., Patai R., Kiss T., Delfavero J., Nagaraja R.Y., Balasubramanian P., Shanmugarama S., Ungvari A. (2025). Senescent Endothelial Cells in Cerebral Microcirculation Are Key Drivers of Age-Related Blood–Brain Barrier Disruption, Microvascular Rarefaction, and Neurovascular Coupling Impairment in Mice. Aging Cell.

[39] Sekeres M.J., Bradley-Garcia M., Martinez-Canabal A., Winocur G. (2021). Chemotherapy-Induced Cognitive Impairment and Hippocampal Neurogenesis: A Review of Physiological Mechanisms and Interventions. Int. J. Mol. Sci..

[40] Wang S., Prizment A., Thyagarajan B., Blaes A. (2021). Cancer Treatment-Induced Accelerated Aging in Cancer Survivors: Biology and Assessment. Cancers.

[41] Wang S., El Jurdi N., Thyagarajan B., Prizment A., Blaes A.H. (2024). Accelerated Aging in Cancer Survivors: Cellular Senescence, Frailty, and Possible Opportunities for Interventions. Int. J. Mol. Sci..

[42] Ma Y., Erb M.L., Moore D.J. (2025). Aging, Cellular Senescence and Parkinson’s Disease. J. Park. Dis..

[43] Alhowail A.H., Aldubayan M. (2021). Recent Progress in the Elucidation of the Mechanisms of Chemotherapy-Induced Cognitive Impairment. Eur. Rev. Med. Pharmacol. Sci..

[44] Williams A.L.M., Phillips N.S., Dong Q., Ehrhardt M.J., Gilmore N., Loh K.P., Meng X., Ness K.K., Hudson M.M., Robison L.L. (2025). Epigenetic Age Acceleration, Telomere Length, and Neurocognitive Function in Long-Term Survivors of Childhood Cancer. Nat. Commun..

[45] Pérez R.F., Tejedor J.R., Santamarina-Ojeda P., Martínez V.L., Urdinguio R.G., Villamañán L., Candiota A.P., Sarró N.M.V., Barradas M., Fernandez-Marcos P.J. (2021). Conservation of Aging and Cancer Epigenetic Signatures across Human and Mouse. Mol. Biol. Evol..

[46] Sehl M.E., Carroll J.E., Horvath S., Bower J.E. (2020). The Acute Effects of Adjuvant Radiation and Chemotherapy on Peripheral Blood Epigenetic Age in Early Stage Breast Cancer Patients. npj Breast Cancer.

[47] Guida J.L., Ahles T.A., Belsky D., Campisi J., Cohen H.J., DeGregori J., Fuldner R., Ferrucci L., Gallicchio L., Gavrilov L. (2019). Measuring Aging and Identifying Aging Phenotypes in Cancer Survivors. JNCI J. Natl. Cancer Inst..

[48] Fulop T., Larbi A., Pawelec G., Khalil A., Cohen A.A., Hirokawa K., Witkowski J.M., Franceschi C. (2023). Immunology of Aging: The Birth of Inflammaging. Clin. Rev. Allergy Immunol..

[49] Behranvand N., Nasri F., Zolfaghari Emameh R., Khani P., Hosseini A., Garssen J., Falak R. (2022). Chemotherapy: A Double-Edged Sword in Cancer Treatment. Cancer Immunol. Immunother..

[50] Gallicchio L., Guida J.L., Green P.A. (2024). Introduction to the Special Section on Cancer Survivors and Treatment-Related Accelerated Aging. J. Cancer Surviv..

[51] Abraham S., Parekh J., Lee S., Afrin H., Rozenblit M., Blenman K.R.M., Perry R.J., Ferrucci L.M., Liu J., Irwin M.L. (2025). Accelerated Aging in Cancer and Cancer Treatment: Current Status of Biomarkers. Cancer Med..

[52] Gaman A.M., Uzoni A., Popa-Wagner A., Andrei A., Petcu E.B. (2016). The Role of Oxidative Stress in Etiopathogenesis of Chemotherapy Induced Cognitive Impairment (CICI)-“Chemobrain”. Aging Dis..

[53] Moruno-Manchon J.F., Uzor N.E., Kesler S.R., Wefel J.S., Townley D.M., Nagaraja A.S., Pradeep S., Mangala L.S., Sood A.K., Tsvetkov A.S. (2018). Peroxisomes Contribute to Oxidative Stress in Neurons during Doxorubicin-Based Chemotherapy. Mol. Cell. Neurosci..

[54] Budamagunta V., Kumar A., Rani A., Manohar Sindhu S., Yang Y., Zhou D., Foster T.C. (2024). Senolytic Treatment Alleviates Doxorubicin-Induced Chemobrain. Aging Cell.

[55] Shachar S.S., Deal A.M., Reeder-Hayes K.E., Nyrop K.A., Mitin N., Anders C.K., Carey L.A., Claire Dees E., Jolly T.A., Kimmick G.G. (2020). Effects of Breast Cancer Adjuvant Chemotherapy Regimens on Expression of the Aging Biomarker, P16^INK4a^. JNCI Cancer Spectr..

[56] Nikolaeva A., Pospelova M., Krasnikova V., Makhanova A., Tonyan S., Krasnopeev Y., Kayumova E., Vasilieva E., Efimtsev A., Levchuk A. (2023). Elevated Levels of Serum Biomarkers Associated with Damage to the CNS Neurons and Endothelial Cells Are Linked with Changes in Brain Connectivity in Breast Cancer Patients with Vestibulo-Atactic Syndrome. Pathophysiology.

[57] Nikolaeva A.T., Pospelova M.L., Krasnikova V.V., Makhanova A.M., Tonyan S.N., Fionik O.V., Efimtsev A.Y., Levchuk A.G., Krasnopeev Y.I. (2023). Clinical and Neuroimaging Laboratory Possibilities of Diagnostics of Vestibulo-Atactic Syndrome in Patients with Postmastectomic Syndrome. Transl. Med..

[58] Pospelova M., Krasnikova V., Fionik O., Alekseeva T., Samochernykh K., Ivanova N., Trofimov N., Vavilova T., Vasilieva E., Topuzova M. (2022). Adhesion Molecules ICAM-1 and PECAM-1 as Potential Biomarkers of Central Nervous System Damage in Women Breast Cancer Survivors. Pathophysiology.

[59] Patel C., Glytsou C., Jang M.H., Cole P.D. (2025). The Effects of Doxorubicin on Blood-Brain Barrier Integrity in HCMEC/D3. Neurotoxicology.

[60] Trudeau J., Ng D.Q., Sayer M., Tan C.J., Ke Y., Chan R.J., Chan A. (2025). Brain-Derived Neurotrophic Factor and Cytokines as Predictors of Cognitive Impairment in Adolescent and Young Adult Cancer Patients Receiving Chemotherapy: A Longitudinal Study. BMC Cancer.

[61] Ng D.Q., Chan D., Agrawal P., Zhao W., Xu X., Acharya M., Chan A. (2022). Evidence of Brain-Derived Neurotrophic Factor in Ameliorating Cancer-Related Cognitive Impairment: A Systematic Review of Human Studies. Crit. Rev. Oncol. Hematol..

[62] Sayer M., Ng D.Q., Trudeau J., Chan R.J., Acharya M.M., Kober K., Chan A. (2025). Epigenetic Age Acceleration and Neurotrophin Signaling Pathways in Cancer-Related Cognitive Impairment: A Longitudinal, Prospective Cohort Study. Front. Aging.

[63] Ng D.Q., Hudson C., Nguyen T., Gupta S.K., Koh Y.Q., Acharya M.M., Chan A. (2025). Dynamin-1 Is a Potential Mediator in Cancer-Related Cognitive Impairment. Neurotherapeutics.

[64] Pospelova M., Krasnikova V., Fionik O., Alekseeva T., Samochernykh K., Ivanova N., Trofimov N., Vavilova T., Vasilieva E., Topuzova M. (2022). Potential Molecular Biomarkers of Central Nervous System Damage in Breast Cancer Survivors. J. Clin. Med..

[65] Claessens A., Manchart A., Boufraine M., Guignard A., Bergeot A., Kieffer A., Lambert A. (2025). Neuron-Specific Enolase as a Biomarker in Ifosfamide-Induced Encephalopathy: A Case Report. Case Rep. Oncol..

[66] Hadjiagapiou M.S., Krashias G., Christodoulou C., Pantzaris M., Lambrianides A. (2023). Serum Reactive Antibodies against the N-Methyl-D-Aspartate Receptor NR2 Subunit—Could They Act as Potential Biomarkers?. Int. J. Mol. Sci..

[67] Ren X., Keeney J.T.R., Miriyala S., Noel T., Powell D.K., Chaiswing L., Bondada S., St. Clair D.K., Butterfield D.A. (2019). The Triangle of Death of Neurons: Oxidative Damage, Mitochondrial Dysfunction, and Loss of Choline-Containing Biomolecules in Brains of Mice Treated with Doxorubicin. Advanced Insights into Mechanisms of Chemotherapy Induced Cognitive Impairment (“chemobrain”) involving TNF-α. Free Radic. Biol. Med..

[68] Murillo L.C., Sutachan J.J., Albarracín S.L. (2023). An Update on Neurobiological Mechanisms Involved in the Development of Chemotherapy-Induced Cognitive Impairment (CICI). Toxicol. Rep..

[69] Demos-Davies K., Lawrence J., Rogich A., Lind E., Seelig D. (2023). Cancer Treatment Induces Neuroinflammation and Behavioral Deficits in Mice. Front. Behav. Neurosci..

[70] McGinnis G.J., Friedman D., Young K.H., Torres E.R.S., Thomas C.R., Gough M.J., Raber J. (2016). Neuroinflammatory and Cognitive Consequences of Combined Radiation and Immunotherapy in a Novel Preclinical Model. Oncotarget.

[71] Demos-Davies K., Lawrence J., Coffey J., Morgan A., Ferreira C., Hoeppner L.H., Seelig D. (2024). Longitudinal Neuropathological Consequences of Extracranial Radiation Therapy in Mice. Int. J. Mol. Sci..

[72] Feiock C., Yagi M., Maidman A., Rendahl A., Hui S., Seelig D. (2016). Central Nervous System Injury—A Newly Observed Bystander Effect of Radiation. PLoS ONE.

[73] Turnquist C., Harris B.T., Harris C.C. (2020). Radiation-Induced Brain Injury: Current Concepts and Therapeutic Strategies Targeting Neuroinflammation. Neurooncol. Adv..

[74] Li M., Tong F., Wu B., Dong X. (2024). Radiation-Induced Brain Injury: Mechanistic Insights and the Promise of Gut–Brain Axis Therapies. Brain Sci..

[75] Constanzo J., Midavaine É., Fouquet J., Lepage M., Descoteaux M., Kirby K., Tremblay L., Masson-Côté L., Geha S., Longpré J.M. (2020). Brain Irradiation Leads to Persistent Neuroinflammation and Long-Term Neurocognitive Dysfunction in a Region-Specific Manner. Prog. Neuropsychopharmacol. Biol. Psychiatry.

[76] Greene-Schloesser D., Robbins M.E., Peiffer A.M., Shaw E.G., Wheeler K.T., Chan M.D. (2012). Radiation-Induced Brain Injury: A Review. Front. Oncol..

[77] Li X., Ding Z. (2024). Cognitive Dysfunction Induced by Cranial Radiotherapy: Mechanisms and Therapeutic Methods. Brain Res. Bull..

[78] Sterpi A.E., Triantafyllou A.S., Tzanetakos D., Ampantzi E., Kitsos D., Theodorou A., Koutsouraki E., Maili M., Stefanou M.I., Moschovos C. (2024). Multiple Sclerosis-like Lesions Induced by Radiation: A Case Report and Systematic Review of the Literature. J. Clin. Med..

[79] Kim J.H., Brown S.L., Gordon M.N. (2023). Radiation-Induced Senescence: Therapeutic Opportunities. Radiat. Oncol..

[80] Markarian M., Krattli R.P., Baddour J.D., Alikhani L., Giedzinski E., Usmani M.T., Agrawal A., Baulch J.E., Tenner A.J., Acharya M.M. (2021). Glia-Selective Deletion of Complement C1q Prevents Radiation-Induced Cognitive Deficits and Neuroinflammation. Cancer Res..

[81] Krattli R.P., Do A.H., El-Khatib S.M., Alikhani L., Markarian M., Vagadia A.R., Usmani M.T., Madan S., Baulch J.E., Clark R.J. (2026). C5aR1 Inhibition Alleviates Cranial Radiation-Induced Cognitive Decline. Cancer Res..

[82] Joly F., Giffard B., Rigal O., De Ruiter M.B., Small B.J., Dubois M., Lefel J., Schagen S.B., Ahles T.A., Wefel J.S. (2015). Impact of Cancer and Its Treatments on Cognitive Function: Advances in Research From the Paris International Cognition and Cancer Task Force Symposium and Update Since 2012. J. Pain Symptom Manag..

[83] Chen W., Hu X., Yao S., Bi Z., Chen M., Cheng H. (2025). Relationship Between Cognitive Disorder and First-Line Targeted Therapy for Oncogene Driver-Positive Patients With Non-Small Cell Lung Cancer: Prospective Cohort Study. JMIR Cancer.

[84] Mayford M. (2007). Protein Kinase Signaling in Synaptic Plasticity and Memory. Curr. Opin. Neurobiol..

[85] Hyland K.A., Eisel S.L., Hoogland A.I., Root J.C., Bowles K., James B., Nelson A.M., Booth-Jones M., Jacobsen P.B., Ahles T.A. (2022). Cognition in Patients Treated with Targeted Therapy for Chronic Myeloid Leukemia: A Controlled Comparison. Leuk. Lymphoma.

[86] Chamoun K., Rabinovich E., Baer L., Fastenau P., de Lima M. (2020). A Case of Neurocognitive Deficit Strongly Related to Dasatinib Therapy. Hematol. Transfus. Cell Ther..

[87] Jones A., Bonomi S., Stockerl-Goldstein K.E., Bateman R.J. (2025). Neuropsychiatric Symptoms Mimicking Dementia in a Patient Treated With Imatinib. Ann. Clin. Transl. Neurol..

[88] Gust J., Hay K.A., Hanafi L.A., Li D., Myerson D., Gonzalez-Cuyar L.F., Yeung C., Liles W.C., Wurfel M., Lopez J.A. (2017). Endothelial Activation and Blood-Brain Barrier Disruption in Neurotoxicity after Adoptive Immunotherapy with CD19 CAR-T Cells. Cancer Discov..

[89] Lee D.W., Santomasso B.D., Locke F.L., Ghobadi A., Turtle C.J., Brudno J.N., Maus M.V., Park J.H., Mead E., Pavletic S. (2018). ASBMT Consensus Grading for Cytokine Release Syndrome and Neurological Toxicity Associated with Immune Effector Cells. Biol. Blood Marrow Transplant..

[90] García-Sánchez J., Torregrosa M.D., Cauli O. (2021). Cognitive Functions under Anti-HER2 Targeted Therapy in Cancer Patients: A Scoping Review. Endocr. Metab. Immune Disord. Drug Targets.

[91] Stangler L.T.B., de Almeida Robatto A.A., Freire P.J.G., de Castro Junior G. (2025). The Challenge of Chemotherapy-Related Cognitive Impairment: Assessing and Managing Cognitive Decline after Cancer Treatment. Ecancermedicalscience.

[92] Das A., Ranadive N., Kinra M., Nampoothiri M., Arora D., Mudgal J. (2020). An Overview on Chemotherapy-Induced Cognitive Impairment and Potential Role of Antidepressants. Curr. Neuropharmacol..

[93] Yi J.C., Syrjala K.L. (2017). Anxiety and Depression in Cancer Survivors. Med. Clin. N. Am..

[94] Perez-Tejada J., Labaka A., Vegas O., Larraioz A., Pescador A., Arregi A. (2021). Anxiety and Depression after Breast Cancer: The Predictive Role of Monoamine Levels. Eur. J. Oncol. Nurs..

[95] Bock K., Peltzer J., Liu W., Colgrove Y., Smirnova I., Siengsukon C. (2025). Sleep Quality and Lymphedema in Breast Cancer Survivors: A Mixed Method Analysis. J. Cancer Surviv..

[96] Noal S., Levy C., Hardouin A., Rieux C., Heutte N., Ségura C., Collet F., Allouache D., Switsers O., Delcambre C. (2011). One-Year Longitudinal Study of Fatigue, Cognitive Functions, and Quality of Life after Adjuvant Radiotherapy for Breast Cancer. Int. J. Radiat. Oncol. Biol. Phys..

[97] Müller J., Ringhof S., Vollmer M., Jäger L.B., Stein T., Weiler M., Wiskemann J. (2020). Out of Balance—Postural Control in Cancer Patients before and after Neurotoxic Chemotherapy. Gait Posture.

[98] Piper K.S., Myhre K.K., Jensen H.E., Madsen K., Mikkelsen M.K., Lund C. (2024). Dizziness and Impaired Walking Balance in Aging Patients during Chemotherapy. J. Geriatr. Oncol..

[99] Simó M., Rifà-Ros X., Rodriguez-Fornells A., Bruna J. (2013). Chemobrain: A Systematic Review of Structural and Functional Neuroimaging Studies. Neurosci. Biobehav. Rev..

[100] Wefel J.S., Vardy J., Ahles T., Schagen S.B. (2011). International Cognition and Cancer Task Force Recommendations to Harmonise Studies of Cognitive Function in Patients with Cancer. Lancet Oncol..

[101] Lange M., Joly F., Vardy J., Ahles T., Dubois M., Tron L., Winocur G., De Ruiter M.B., Castel H. (2019). Cancer-Related Cognitive Impairment: An Update on State of the Art, Detection, and Management Strategies in Cancer Survivors. Ann. Oncol..

[102] Bai L., Yu E. (2021). A Narrative Review of Risk Factors and Interventions for Cancer-Related Cognitive Impairment. Ann. Transl. Med..

[103] Fernandes H.A., Richard N.M., Edelstein K. (2019). Cognitive Rehabilitation for Cancer-Related Cognitive Dysfunction: A Systematic Review. Support. Care Cancer.

[104] Vardy J.L., Pond G.R., Bell M.L., Renton C., Dixon A., Dhillon H.M. (2022). A Randomised Controlled Trial Evaluating Two Cognitive Rehabilitation Approaches for Cancer Survivors with Perceived Cognitive Impairment. J. Cancer Surviv..

[105] Campbell K.L., Zadravec K., Bland K.A., Chesley E., Wolf F., Janelsins M.C. (2020). The Effect of Exercise on Cancer-Related Cognitive Impairment and Applications for Physical Therapy: Systematic Review of Randomized Controlled Trials. Phys. Ther..

[106] Sánchez S.J., Gómez C.S., Gutiérrez S.S., García-Tizón S.J., González J.L.S., Galve M.I.R., Sánchez E.F., Rodríguez E.J.F. (2025). A Cognitive Training Programme on Cancer-Related Cognitive Impairment (CRCI) in Breast Cancer Patients Undergoing Active Treatment: A RCT Study Protocol. J. Clin. Med..

[107] Kiesl D., Kuzdas-Sallaberger M., Fuchs D., Brunner S., Kommenda R., Tischler C., Hornich H., Akbari K., Kellermair J., Blessberger H. (2022). Protocol for the Exercise, Cancer and Cognition—The ECCO-Study: A Randomized Controlled Trial of Simultaneous Exercise During Neo-/Adjuvant Chemotherapy in Breast Cancer Patients and Its Effects on Neurocognition. Front. Neurol..

[108] Gates P., Green H.J., Gough K., Dhillon H., Vardy J.L., Dickinson M., Guarnera J., Krishnasamy M., Livingston P.M., White V. (2024). Web-Based Cognitive Rehabilitation Intervention for Cancer-Related Cognitive Impairment Following Chemotherapy for Aggressive Lymphoma: Protocol for a Randomised Pilot Trial. BMJ Open.

[109] Rapp S.R., Dressler E.V.M., Brown W.M., Wade J.L., Le-Lindqwister N., King D.M., Rowland K.M., Weaver K.E., Klepin H.D., Shaw E.G. (2023). Phase 3 Randomized Placebo-Controlled Trial of Donepezil for Late Cancer-Related Cognitive Impairment in Breast Cancer Survivors Exposed to Chemotherapy from the Wake Forest NCORP Research Base REMEMBER Trial (WF97116). J. Clin. Oncol..

[110] Henneghan A., Rao V., Harrison R.A., Karuturi M., Blayney D.W., Palesh O., Kesler S.R. (2020). Cortical Brain Age from Pre-Treatment to Post-Chemotherapy in Patients with Breast Cancer. Neurotox. Res..

[111] Daniel E., Deng F., Patel S.K., Sedrak M.S., Kim H., Razavi M., Sun C.-L., Root J.C., Ahles T.A., Dale W. (2023). Altered Gyrification in Chemotherapy-Treated Older Long-Term Breast Cancer Survivors. Res. Sq..

[112] Mohammadi M., Banisharif S., Moradi F., Zamanian M., Tanzifi G., Ghaderi S. (2024). Brain Diffusion MRI Biomarkers after Oncology Treatments. Rep. Pract. Oncol. Radiother..

[113] Alexander S., Nikolaeva A., Pospelova M., Voynov M., Krasnikova V., Makhanova A., Tonyan S., Efimtsev A., Olga F., Levchuk A. (2025). Diffusion Tensor Tractography Shows White Matter Tract Changes in Breast Cancer Survivors with Balance Impairment. Pathophysiology.

[114] Kim H.S., Jung C.O., Jeon H.R., Sung L.H. (2012). Rehabilitation for Ataxia Following Chemotherapy for Burkitt Lymphoma Involving the Rectum. Ann. Rehabil. Med..

[115] Land S.R., Kopec J.A., Cecchini R.S., Ganz P.A., Wieand H.S., Colangelo L.H., Murphy K., Kuebler J.P., Seay T.E., Needles B.M. (2007). Neurotoxicity from Oxaliplatin Combined with Weekly Bolus Fluorouracil and Leucovorin as Surgical Adjuvant Chemotherapy for Stage II and III Colon Cancer: NSABP C-07. J. Clin. Oncol..

[116] Magge R.S., DeAngelis L.M. (2015). The Double-Edged Sword: Neurotoxicity of Chemotherapy. Blood Rev..

[117] Chen B.T., Chen Z., Deng F., Patel S.K., Sedrak M.S., Root J.C., Ahles T.A., Razavi M., Kim H., Sun C.L. (2022). Signal Variability and Cognitive Function in Older Long-Term Survivors of Breast Cancer with Exposure to Chemotherapy: A Prospective Longitudinal Resting-State FMRI Study. Brain Sci..

[118] Bukkieva T., Pospelova M., Efimtsev A., Fionik O., Alekseeva T., Samochernych K., Gorbunova E., Krasnikova V., Makhanova A., Levchuk A. (2022). Functional Network Connectivity Reveals the Brain Functional Alterations in Breast Cancer Survivors. J. Clin. Med..

[119] Nikolaeva A., Pospelova M., Krasnikova V., Makhanova A., Tonyan S., Efimtsev A., Levchuk A., Trufanov G., Voynov M., Sklyarenko M. (2025). MRI Voxel Morphometry Shows Brain Volume Changes in Breast Cancer Survivors: Implications for Treatment. Pathophysiology.

[120] Hatchard T., Penta S., Mioduzsewski O., Correia S., Tissera T., Brown O., Haefner S.A., Poulin P., Smith A.M. (2022). Increased Gray Matter Following Mindfulness-Based Stress Reduction in Breast Cancer Survivors with Chronic Neuropathic Pain: Preliminary Evidence Using Voxel-Based Morphometry. Acta Neurol. Belg..

